# DNA barcoding and biomass accumulation rates of native Iranian duckweed species for biotechnological applications

**DOI:** 10.3389/fpls.2022.1034238

**Published:** 2022-11-29

**Authors:** Elham Taghipour, Manuela Bog, Fateme Frootan, Sadegh Shojaei, Nima Rad, Mahdi Arezoumandi, Mahyat Jafari, Ali Hatef Salmanian

**Affiliations:** ^1^ National Institute of Genetic Engineering and Biotechnology (NIGEB), Department of Agricultural Biotechnology, Tehran, Iran; ^2^ University of Greifswald, Institute of Botany and Landscape Ecology, Greifswald, Germany

**Keywords:** duckweed, *Lemna*, RGR, DNA barcoding, biomass accumulation, doubling time, biotechnology, food security

## Abstract

The Lemnaceae family (duckweed) consists of at least three recognized genera with six reported species in Iran that are distributed in wetlands. Duckweeds are the simplest and smallest flowering aquatic monocots with free-floating fronds that can reproduce asexually every 2–3 days. Duckweed could be a major source of balanced amino acids and high protein content, which is increasingly promising for biotechnological applications. For molecular classification and species identification of the collected samples, DNA barcoding was performed using two standard chloroplast markers, the spacer region between the ATP synthase subunits F and H (*atpF-atpH*) and the intron region of the ribosomal protein S16 (*rps16*). The results confirm the presence of four species belonging to the two genera *Lemna* and *Spirodela*. In addition, *L. turionifera* was detected for the first time in Iran. Due to the high growth rates of duckweed, measurement of biomass accumulation and doubling time are important factors in determining growth potential, especially for native species. The relative growth rates (RGR), doubling times (DT), biomass accumulation, and relative weekly yields (RY) of 40 distinct duckweed clones were determined under standard cultivation conditions. The dry weight–based RGR ranged from 0.149 to more than 0.600 per day, DT from 1.12 to 9 days, and RY from 7 to 108.9 per week. All values are comparable with previous studies. RGR and RY of selected clones are higher than the growth potential for a wide range of wild plants and common crops. These data support that native duckweed has high productivity value and should be further investigated as a potentially rich protein source for alternative human food, livestock feed, and recombinant protein production.

## Introduction

The cosmopolitan monocotyledonous family Lemnaceae (duckweed), which includes the smallest and fastest growing angiosperms known, comprises 36 species in five genera: *Spirodela* Schleid., *Landoltia* Les & Crawford, *Lemna* L., *Wolffia* Horkel ex Schleid., and *Wolffiella* Hegelm. (Bog et al., 2020). However, some authors claim that duckweeds should be reclassified as subfamily Lemnoideae in the Araceae family based on a close phylogenetic relationship. However, the inclusion of duckweed in the Araceae would remove a useful and well-defined taxonomic category of an angiosperm family that has been used by duckweed biologists for many years (Tippery et al., 2021). These tiny aquatic plants grow on or below the surface of slow-flowing, nutrient-enriched water bodies. Their morphology is highly reduced to simple leaf-like structures also known as fronds that appear genus-specific with or without roots. Duckweeds can create genetically uniform populations by their rapid vegetative propagation. These properties, their small size, rapid and high yield growth, and additionally relatively small genome sizes make duckweeds an ideal experimental material and a powerful platform for various biotechnological applications (Appenroth et al., 2013; Heenatigala et al., 2020; Tippery et al., 2021). Under optimized growth conditions, duckweed contains a high protein content of up to 45% with high-quality and easily digestible amino acids close to the recommendations of the World Health Organization (WHO), which is an important nutritional index (Mes et al., 2022a; Pagliuso et al., 2022). Therefore, there is increased interest in the use of duckweed species from the genera *Wolffia* and *Lemna* as a good protein source, particularly for use in human food and animal nutrition (Edelman and Colt, 2016; Appenroth et al., 2017; Appenroth et al., 2018). Moreover, the high fiber content (∼25% of dry weight) and polyunsaturated fatty acids (more than 60% of total fat) are shown to be a unique nutrient composition of duckweed (Appenroth et al., 2018). While major crops such as rice, maize, and wheat often have an imbalance in nutrient composition, the amino acids, vitamins, mineral profiles, and fatty acid fractions of duckweed have a high-quality composition (Edelman and Colt, 2016). The increase in world population, climate change, and decrease in food supply have increased pressure on food systems. In addition, excessive land use and agricultural activities have led to soil erosion, resulting in a 0.4% per year decline in global crop yields (Pennock, 2019). Therefore, there are increasing demands for the development of a sustainable food and feed safety system (Pagliuso et al., 2022). For example, the European Food and Safety Authority considers all genera of duckweed as novel foods (Mes et al., 2022b). Most notably, the ease of cultivation of these tiny aquatic plants in multilayered vertical farming systems and their high tolerance to a wide range of environmental conditions around the world may reduce competition with terrestrial crops (Coughlan et al., 2022; Pagliuso et al., 2022).

In addition, duckweed can be used in phytoremediation, water quality measurement, and wastewater treatment. Duckweeds have the ability to accumulate macronutrients and micronutrients hundreds of times compared with the mineral concentration of the water in which they proliferate (Chakrabarti et al., 2018; Walsh et al., 2021). The high biomass accumulation with a typically high protein content between 24% and 45% makes duckweed a suitable supplement for animal feed (Iatrou et al., 2015; Pagliuso et al., 2022). Further, duckweed can be used for bioethanol production due to its high starch accumulation under stress conditions (Sree and Appenroth, 2014; Liu et al., 2019).

The potential application of duckweed in plant bioreactors has attracted increasing attention due to its rapid doubling time (DT) and proliferation of uniform clones with nearly exponential growth (Edelman et al., 2020). Their growth rate is nearly 28 times faster than conventional crops used for human nutrition (Pagliuso et al., 2022; Coughlan et al., 2022). Undoubtedly, this higher reproductive rate will significantly shorten the production cycle of duckweed in bioreactors, leading to a maximum biomass accumulation of up to 100 tons of dry matter per hectare per year (Cao et al., 2018). Therefore, the study of biomass production and growth factors in duckweed under different cultivation conditions could be interesting. There is a number of pilot studies on duckweed biomass production under environmental conditions where wastewater or enriched medium is used to grow duckweed. The reports describe high-yield biomass production of 8 t dw/ha/y for *Wolffia arrhiza* (L.) Horkel ex Wimm. (Fujita et al., 1999) and 36 t dw/ha/y for *Spirodela polyrhiza* (L.) Schleid. (Xu et al., 2012) and up to 104 t/ha/year for *Lemna minor* L. (Frederic et al., 2006), which is comparable to the average yields of major land crops reported by the Food and Agriculture Organization of the United Nations (FAO, 2013) and the U.S. Department of Agriculture (USDA) (Ziegler et al., 2015).

The quality value of duckweed as a food source depends on the content and composition of constituents, particularly amino acid profiles, protein content, and high potential for rapid growth. Therefore, cultivation conditions and especially the genetic background of ecotypes are assumed to play a crucial role (Appenroth et al., 2018; Chakrabarti et al., 2018). Thus, assuming that different geographic isolates (ecotypes) of duckweeds have genetically differentiated due to adaptation to specific environmental conditions, the resulting clones may exhibit different physiological and growth behavior (Ziegler et al., 2015; Chakrabarti et al., 2018; Walsh et al., 2021). Studying a wide range of these ecotypes around the world may lead to the identification of superior clones in terms of growth characteristics that can be eligible for other studies in different fields, such as biochemical analysis to introduce an alternative food supply.

To date, only a limited number of extensive studies have been conducted to investigate growth factors and biomass production in duckweed ecotypes from different parts of the world. Bergmann et al. (2000) studied 41 geographic isolates of 12 species from Landolt’s worldwide stock collection at ETH Zurich (now hosted by the Istituto di Biologia e Biotecnologia Agraria in Milano, Italy) under *in vitro* conditions in a synthetic medium. To assess growth, they reported only wet weight gain and the percentage dry weight during the 11-day growth period for selection of superior geographic isolates (Bergmann et al., 2000). Among others, the relative growth rate (RGR) is an important growth factor that reflects the growth potential, especially in duckweeds. A comprehensive study of the RGR of duckweed was already carried out by Landolt (1957), who investigated 71 clones of 13 species. Ziegler et al. (2015) presented two other growth factors in addition to RGR to provide comparable data with other reports, e.g., on terrestrial crops. RGR, DT, and relative weekly yield (RY) of 39 ecotypes from 13 duckweed species were determined under standard cultivation conditions using a modified Schenk–Hildebrand medium for 7 days. Here, the mean RGR was 0.304 per day for *Spirodela* and 0.396 per day for *Lemna*. In general, RGR ranged from 0.153 to 0.519 per day, DT from 1.34 to 4.54 days, and RY from 2.9 to 37.8 per week for the duckweed species studied (Ziegler et al., 2015). Sree et al. (2015) investigated the RGR of 25 clones representing all 11 species of the genus *Wolffia*, the genus commonly used for human nutrition. They present a clone of *Wolffia microscopica* (Griff.) Kurz with a doubling time of 29.3 h, which is the fastest growing flowering plant (Sree et al., 2015). Other reports determining the RGR of duckweed species have reported an RGR of 0.31 per day for *Lemna minor* and 0.30 and 0.42 per day for *Lemna gibba* (Lasfar et al., 2007).

One of the first important steps is the precise identification of plant species. Several methods, such as morphological, biochemical, and molecular comparisons, can be used for correct identification. Among the mentioned marker types, comparison of molecular data with references is the most reliable (Dogan et al., 2014; Hasanbegovic et al., 2021; Saran et al., 2021). Specifically for duckweed, identification using only morphological characteristics is nearly impossible even for experts because the morphological structure of duckweed is greatly reduced. Using molecular methods, such as DNA barcoding, which is based on DNA markers, it is possible to reproducibly and reliably identify most duckweed species (Borisjuk et al., 2015; Bog et al., 2019). To the best of our knowledge, the native duckweeds of Iran have not been studied at the molecular level or in terms of growth rate. This is the first report on DNA barcoding of the only duckweed collection in Iran.

In the present study, RGR, DT, and RY for duckweed species native to Iran are investigated for the first time. Most of the clones studied are from the north of Iran, where most of the duckweed habitats are located. The investigation of 40 Iranian clones, which can be assigned to four species within the two genera *Lemna* and *Spirodela*, aims to determine accumulation of biomass yields and to identify the superior ecotypes with high growth potential under laboratory conditions for future biotechnological applications and food safety research.

## Material and methods

### Plant material

Duckweed samples were collected from different natural ponds in the north of Iran (Mazandaran and Gilan provinces) and a region in the west of Iran (Kermanshah province). The geographical distribution map of sampling can be found in **Supplementary Figure 1**. Fronds were rinsed in clean tap water and sterilized using 2.5% sodium hypochlorite solution for 1 min and subsequently washed three times with sterile distilled water. Sterilized fronds of all *Lemna* species were then cultivated in modified Hoagland medium (Khvatkov et al., 2019) except *L. gibba*, which was cultivated in NF medium (Muranaka et al., 2015). Schenk–Hildebrand medium (Schenk and Hildebrandt, 1972) was used for the *Spirodela* species. All media were supplemented with 1% sucrose. All clones were cultivated under standard cultivation conditions (ISO 20079, 2005) with a 16/8 h light/dark photoperiod with 80 μmol/m^2^/s light intensity from fluorescent light tubes (40W) (Pars shahab, Tehran, Iran) at 25°C.

### Morphological identification and molecular analysis

Duckweed samples were primarily identified morphologically using the key from Riedl (1976) and the updated key from Bog et al. (2020) based on frond shape, frond size, and number of roots and veins. The identity of the clones that were chosen for the biomass accumulation test was confirmed by DNA barcoding. For molecular analysis, total DNA was extracted by a modified CTAB protocol (Murray and Thompson, 1980). The chloroplast marker from the noncoding spacer *atpF-atpH* was amplified using the primers *atpF-atpH* forward (5’ ACTCGCACACACTCCCTTTCC 3’) and *atpF-atpH* reverse (5’ GCTTTTATGGAAGCTTTAACAAT 3’) as described previously (Wang et al., 2010). The PCR conditions were predenaturation at 94°C for 2 min, followed by 35 cycles of 94°C, 15 s; 51°C, 15 s; 72°C, 40 s; and a final extension at 72°C for 5 min. The primer set of the second marker, *rps16*, was used to amplify the chloroplast ribosomal protein S16 gene intron with the degenerate primers *rps16* F (5’ AAACGATGTGGTARAAAGCAAC 3’) and *rps16* R (5’ AACATCWATTGCAASGATTCGATA 3’) as described previously (Shaw et al., 2005). The PCR conditions were predenaturation at 94°C for 5 min, followed by 45 cycles at 94°C, 30 s; 61°C, 50 s; 72°C, 80s; and a final extension at 72°C for 7 min. The PCR fragments were purified and further processed for sequencing by the Beijing Genomic Institute (BGI, Shenzhen, China). Sequences were deposited in GenBank (https://www.ncbi.nlm.nih.gov/). Accession numbers of the *rps16* and *atpF-atpH* sequences are listed in **Table 1**.

**Table 1:** Duckweed species investigated for this study with their NCBI accession numbers.

Row	Species	Strain	Origin	Accession number	
				*atpF-atpH*	*rps 16*
1	*Lemna minor*	1C	Tonekabon pond, Iran	MT891062	MW308182
2	*Lemna minor*	2C	Tonekabon pond, Iran	MT891063	MZ422535
3	*Lemna minor*	3a	Tonekabon pond, Iran	MT891064	MW308183
4	*Lemna minor*	4BM	Mansoori pond, Iran	MT891065	MZ422534
5	*Lemna minor*	5C	Tonekabon pond, Iran	MT891066	MW308184
6	*Lemna minor*	6a	Tonekabon pond, Iran	MT891067	MW308185
7	*Lemna minor*	7W	Tonekabon pond, Iran	MT891068	MW308186
8	*Lemna minor*	8C	Tonekabon pond, Iran	MT891069	MW308187
9	*Lemna minor*	9a	Tonekabon pond, Iran	MT891070	MW308188
10	*Lemna minor*	10C	Tonekabon pond, Iran	MT891071	MW308189
11	*Lemna minor*	11C	Tonekabon pond, Iran	MT891072	MW308190
12	*Lemna minor*	12W	Tonekabon pond, Iran	MT891073	MZ422533
13	*Lemna minor*	13C	Tonekabon pond, Iran	MT891074	MW308191
14	*Lemna minor*	14BM	Mansoori pond, Iran	MT891075	MZ422532
15	*Lemna minor*	15C	Tonekabon pond, Iran	MT891076	MW308192
16	*Lemna minor*	16C	Tonekabon pond, Iran	MT891077	MW308193
17	*Lemna turionifera*	17AM	Amir kelaye international lagoon, Iran	MT891086	MW308200
18	*Lemna minor*	18C	Tonekabon pond, Iran	MT891078	MW308194
19	*Lemna minor*	19C	Tonekabon pond, Iran	MT891079	MW308195
20	*Lemna turionifera*	20AM	Amir kelaye international lagoon, Iran	MT891087	MW308201
21	*Lemna minor*	21BM	Mansoori pond, Iran	MT891080	MW308196
22	*Lemna minor*	22a	Tonekabon pond, Iran	MT891081	MW308197
23	*Lemna minor*	23C	Tonekabon pond, Iran	MT891082	MW308198
24	*Lemna minor*	24W	Tonekabon pond, Iran	MT891083	MZ422531
25	*Lemna minor*	25C	Tonekabon pond, Iran	MT891084	MZ422530
26	*Lemna minor*	26W	Tonekabon pond, Iran	MT891085	MW308199
27	*Lemna gibba*	1LA	Langarud paddy,Iran	MT891088	MW308202
28	*Lemna gibba*	2S	Soostan Lagoon, Iran	MT891089	MW308203
29	*Lemna gibba*	3g	Govaver, Iran	MT891092	MW308206
30	*Lemna gibba*	4LA	Langarud paddy, Iran	MT891093	MW308207
31	*Lemna gibba*	5S	Soostan Lagoon, Iran	MT891091	MW308205
32	*Lemna gibba*	6LA	Langarud paddy, Iran	MT891090	MW308204
33	*Lemna gibba*	7S	Soostan Lagoon, Iran	MT891094	MZ422528
34	*Lemna gibba*	8S	Soostan Lagoon, Iran	MT891095	MZ422529
35	*Spirodela polyrhiza*	1AM	Amir kelaye international lagoon, Iran	MT891096	MW308208
36	*Spirodela polyrhiza*	2AM	Amir kelaye international lagoon, Iran	MT891097	MW308209
37	*Spirodela polyrhiza*	3LA	Langarud lagoon, Iran	MT891098	MW308210
38	*Spirodela polyrhiza*	4AM	Amir kelaye international lagoon, Iran	MT891099	MW308211
39	*Spirodela polyrhiza*	5BM	Mansoori pond, Iran	MT891100	MW308212
40	*Spirodela polyrhiza*	6AM	Amir kelaye international lagoon, Iran	MT891101	MZ422536

### DNA barcoding analysis

To analyze the genetic diversity, *atpF-atpH* and *rps16* sequences were checked using Chromas Lite 2.6.2 (Technelysium Pty Ltd, South Brisbane, Australia) and aligned using the BioEdit Sequence Alignment Editor 7.1.3.0 (Hall, 1999). Reference sequences of *atpF-atpH* and *rps16* markers for DNA barcoding were prepared from already identified clones from the duckweed stock collection of the University Greifswald (Germany) or taken from GenBank (**Table 2**). They were chosen to represent a wide geographical distribution of the species. SeqState 1.4.1 (Müller, 2005) was used to recode insertion and deletion (indel) positions using the implemented Simmons and Ochoterena simple coding algorithm, leading to a final alignment length of 798 sites including 14 indel coded sites for *rps16* and a final alignment length of 661 sites including 12 indel coded sites for *atpF-atpH*. The indel coded alignments can be found as **Supplementary Material 1** and **2**. Subsequently, TCS 1.23 (Clement et al., 2000) was used with default settings to build haplotype networks for each chloroplast marker. Based on the haplotype results, the alignments were collapsed to unique haplotypes for which a maximum-likelihood tree was built using iqTREE 2.1.3 (Minh et al., 2020) with 1000 bootstrap replicates. The implemented ModelFinder (Kalyaanamoorthy et al., 2017) chose F81+F (*atpF-atpH*) and K3Pu+F (*rps16*) as the best-fit models according to the Bayesian information criterion. Finally, DnaSP 6.12.03 (Rozas et al., 2017) was run to count polymorphic and parsimony informative sites and to estimate nucleotide and haplotype diversity.

**Table 2:** Reference sequences of *atpF-atpH* and *rps16* markers for DNA barcoding.

Row	Reference sequences	Strain	Origin	Accession number	
				*atpF-atpH*	*rps 16*
1	*Lemna minor*	7123	Canada, Saskachewan, Saskatoon	MG000397	*
2	*Lemna minor*	8292	Iran, Mazanda, Ramsar, Ghassem Abbath	*	*
3	*Lemna minor*	9441	Germany, Marburg (clone St)	*	*
4	*Lemna turionifera*	6573	USA, Montana, Lincoln Co.	MG775403	*
5	*Lemna turionifera*	7683	Korea, Kyonggi, Sosa	MG775404	*
6	*Lemna turionifera*	9434	Russia, Lake Baikal	MG775405	*
7	*Lemna gibba*	7589	USA, California, Los Angeles Co., Covina	GU454219	*
8	*Lemna gibba*	7741	Italy, Sicilia, Siracusa (clone G3)	KX212887	*
9	*Lemna gibba*	8703	Japan, Honshu Aichi	GU454222	*
10	*Spirodela polyrhiza*	7373	Egypt, Mahallet, El Rahabein	HG938145	HG938250
11	*Spirodela polyrhiza*	7498	USA, North Carolina, Durham Co., Durham	GU454204	HG938251
12	*Spirodela polyrhiza*	9500	Germany, Jena, Porstendorf 1967 (clone SJ)	GU454208	HG938257

*sequenced but no Genbank number yet.

### Culture conditions for growth factor analysis

Forty geographic isolates representing four species from two genera were used for biomass accumulation and DT analysis as described in **Table 1**. According to the ISO 20079 protocol (ISO 20079, 2005), the sterilized duckweed samples were precultivated for 1 month to acclimatize the clones to the cultivation conditions. Nutrient media were replenished every week. A single clone with the same frond number from each species was used for initial inoculation of 50 ml nutrient medium in glass jars covered with plastic caps. All glasses were kept under axenic conditions at 25°C in a standard growth chamber. The investigated duckweed species showed optimum growth in different media (unpublished results). For this reason, we used a specific nutrient medium for each species instead of the Steinberg medium specified in the ISO 20079 protocol as mentioned. The growth factors were determined starting with a four-frond colony for each species, and the initial weight was determined. The main cultivation phase lasted 7 days, taking care that the fronds never completely covered the surface of the medium, which may limit growth.

### Calculation of growth parameters

All growth parameters were determined at the onset of the experiment (t_0_) and 7 days later (t7). The number of fronds (FN_0_ and FN_7_), fresh weight (FW_0_ and FW_7_), and dry weight (DW_0_ and DW_7_) were measured. At the initiation of the experiment, the frond numbers were recorded. Then, an equal frond number and size was surface-dried by filter paper and weighed (FW_0_). These reference samples were dried at 37°C for 72 h to determine the dry weight of the preliminary inoculum (DW_0_). Frond number, fresh weight, and dry weight of fronds at t_7_ were determined as for the reference samples from t_0_. Three independent experiments were conducted with each of the clones.

RGR was calculated using Equation (1) (Naumann et al., 2007; Ziegler et al., 2015). This equation was simplified to Equation (2) for better interpretation of growth potentials. The values of measured parameters *x* (Frond number or fresh and dry weight) in two time points (t_0_ and t_7_) were placed in Equation (2).


(1) $$X_{t}=\;x_{t0}\times e^{RGR\;\times \;t}$$

(2) $$RGR\;=\;(lnx_{t7}-\;lnx_{t0})\;/\;\left(t7\;-\;t0\right)$$

The RGR unit is based on time (per day). DT (days) or biomass accumulation (per day), was calculated by Equation (3), when RGR is measured with frond number values or fresh weight and dry weight of fronds at the two time points, respectively.


(3) (BA) DT = ln2 / RGR

The yield obtained from the initial inoculum of one frond (or 1 mg duckweed biomass) after 7 days of cultivation is known as RY. It was calculated using Equation (4):


(4) $$RY={\text{l}n\text{x}}_{t7}=\ln x_{to}+RGR\times (t7-t0)$$

RY is equal to lnx_t7_, and *x* is one of the growth parameters measured in the experiment, such as FN, FW, and DW at t_0_ (lnx_t0_) and at t_7_ (lnx_t7_). The RY of one frond or 1 mg duckweed initial inoculum after 7 days has the unit per week.

### Data analysis

Statistical analysis was carried out in SPSS 16.0 (IBM, USA). The normality of the data was confirmed by the Kolmogorov–Smirnov test. Therefore, parametric methods were used to compare the means. The variation of means among groups was compared with one-way ANOVA using the Student–Newman–Keuls test (SNK), a *post hoc* test for analysis of the differences in means, at the level of *P* ≤.05.

## Results

### DNA barcoding of duckweed ecotypes based on *rps16* and *atpF-atpH* sequences

**Figure 1:**
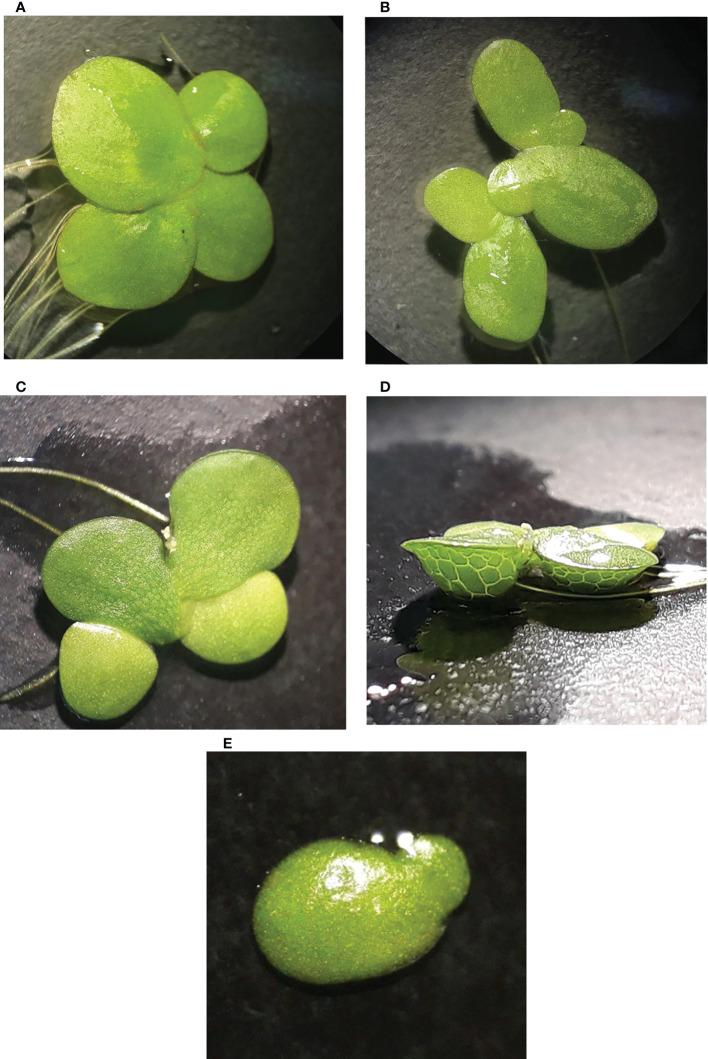
Four investigated duckweed species native to Iran; **(A)** *Spirodela polyrhiza.* **(B)** *Lemna minor.* **(C)** *L. gibba* in dorsal view. **(D)** *L. gibba* in ventral view. **(E)** *L. turionifera*.

A total of 40 duckweed clones were collected from the north of Iran (lakes of Tonekabon, Lahijan, and Langarud) and the Kermanshah Govaver River. The collected duckweed accessions were morphologically determined and validated by molecular methods, i.e., DNA barcoding (**Figure 1**).

In summary, a total of 24 ecotypes of *L. minor*, eight ecotypes of *L. gibba*, six ecotypes of *S. polyrhiza*, and two ecotypes of *L. turionifera*, a rare species for Iran, were identified with the chloroplast fragments *rps16* and *atpF-atpH*. All identified clones were successfully propagated to produce pure clones.

**Figure 2:**
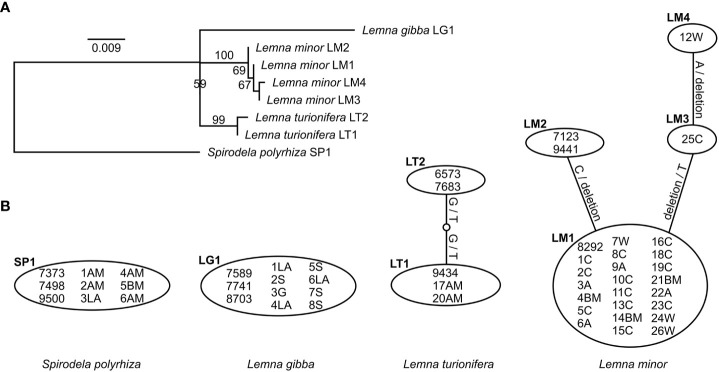
Molecular genetic results of the cp marker *rps16*. **(A)** Maximum-likelihood tree of unique haplotypes found for all investigated clones. Bootstrap values based on 1,000 replicates. *Spirodela polyrhiza* was set as outgroup. Scale indicates number of substitutions per site. **(B)** Identification of haplotypes and their differences for all investigated clones. Substitutions are given on the lines. - denotes deletion.

**Figure 3:**
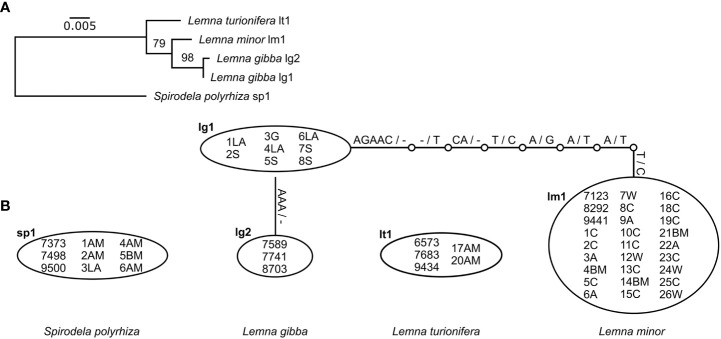
Molecular genetic results of the cp marker *atpF-atpH*. **(A)** Maximum-likelihood tree of unique haplotypes found for all investigated clones. Bootstrap values based on 1,000 replicates. *Spirodela polyrhiza* was set as outgroup. Scale indicates number of substitutions per site. **(B)** Identification of haplotypes and their differences for all investigated clones. Substitutions are given on the lines. - denotes deletion.

The species could be very well-distinguished by both chloroplast markers (*rps16* and *atpF-atpH*) as represented in the maximum-likelihood phylogenetic trees (**Supplementary Figures 2A, B**). Comparison of the two sequence alignments for both markers separately shows that *rps16* has a higher haplotype and nucleotide diversity than *atpF-atpH* although differences between different haplotypes within one species are most often caused by indels (**Table 3**, **Figures 2**, **3**). For *rps16*, both clones identified as *L. turionifera* from Iran showed the same haplotype (LT1) as the reference sequence of clone 9434 from Lake Baikal, Russia. For *L. minor* the Iranian clones were identical to the haplotype (LM1) of 8292, a reference clone from Iran, too. Two further clones (25C – haplotype LM3 and 12W – haplotype LM4) showed one or two additional bases but are more similar to the main haplotype LM1 found for Iran than to the other two reference clones from Canada and Germany (haplotype LM2) (**Figures 2A, B**). For the marker *atpF-atpH* only *L. gibba* revealed different haplotypes, in which the Iranian clones differed by an additional stretch of three A’s (haplotype lg1) from the three reference clones, which showed the same haplotype (lg2) (**Figures 3A, B**).

**Table 3:** Alignment characteristics for the two investigated chloroplast markers.

species	numberclones	*rps16*							*atpF-atpH*						
		alignmentlength (bp)*	indel codedsites	PS	PI	H_num_	H_d_ ± SD	л ± SD	alignmentlength (bp)*	indel codedsites	PS	PI	H_num_	H_d_ ± SD	л ± SD
*S. polyrhiza*	9	798	14	0	0	1	0.000± 0.000	0.0000± 0.0000	661	12	0	0	1	0.000± 0.000	0.0000± 0.0000
*L. gibba*	11			0	0	1	0.000± 0.000	0.0000± 0.0000			1	1	2	0.436± 0.133	0.0008± 0.0002
*L. turionifera*	5			2	2	2	0.600± 0.175	0.0016± 0.0005			0	0	1	0.000± 0.000	0.0000± 0.0000
*L. minor*	27			3	2	4	0.276± 0.109	0.0005± 0.0002			0	0	1	0.000± 0.000	0.0000± 0.0000

PS, polymorphic sites; PI, parsimony informative sites; H_num_, number of haplotypes; H_d_, haplotype diversity; л, nucleotide diversity; SD, standard deviation.* including indel coded sites.

### Biomass accumulation and doubling time

The growth potential measured as RGR and RY based on fresh and dry weight and DT based on frond numbers of 40 geographic duckweed isolates under axenic cultivation conditions are shown in **Table 4**. Overcrowding of populations was not observed during the experiment or at the end after 7 days. This ensured that the growth was not inhibited by intraspecific competition. In addition, axenic cultivation prevented inhibitory effects of undesirable microorganisms (Ziegler et al., 2015). During the 7 days of the experiment, the increase in frond number, fresh weight, and dry weight never deviated from an exponential progression.

**Table 4:** Fresh and dry weight biomass accumulation and doubling time of 40 geographical isolates of native Iranian duckweed representing four species.

Row	Species	strain	DT_FN_	RGR_FW_	RGR_DW_	BA_FW_	BA_DW_	RY_FW_	RY_DW_
1	*Lemna minor*	1C	3.77 ± 0.11	0.337 ± 0.029	0.478 ± 0.01	2.10 ± 0.18	1.51 ± 0.02	28.94 ± 5.55	3.71 ± 0.02
2	*Lemna minor*	2C	3.44 ± 0.12	0.279 ± 0.005	0.255 ± 0.01	2.49 ± 0.05	2.72 ± 0.06	30.68 ± 1.14	2.14 ± 0.09
3	*Lemna minor*	3a	5.97 ± 0.42	0.215 ± 0.001	0.307 ± 0.01	3.10 ± 0.06	2.39 ± 0.02	14.44 ± 0.25	2.05 ± 0.03
4	*Lemna minor*	4BM	3.73 ± 0.001	0.338 ± 0.006	0.277 ± 0.01	2.05 ± 0.03	2.50 ± 0.06	29.69 ± 1.14	1.75 ± 0.09
5	*Lemna minor*	5C	3.67 ± 0.15	0.343 ± 0.002	0.256 ± 0.01	2.02 ± 0.01	2.72 ± 0.11	34.87 ± 0.43	2.05 ± 0.14
6	*Lemna minor*	6a	7.05 ± 0.02 (-)	0.301 ± 0.025	0.287 ± 0.02	2.35 ± 0.20	2.44 ± 0.14	28.21 ± 4.84	1.75 ± 0.2
7	*Lemna minor*	7W	4.04 ± 0.01	0.215 ± 0.001	0.332 ± 0.02	3.23 ± 0.02	2.18 ± 0.03	28.06 ± 0.20	2.94 ± 0.09
8	*Lemna minor*	8C	5.34 ± 0.16	0.202 ± 0.005	0.156 ± 0.01 (-)	3.44 ± 0.08	4.52 ± 0.37 (-)	12.65 ± 0.43	1.30 ± 0.12
9	*Lemna minor*	9a	3.74 ± 0.01	0.277 ± 0.007	0.294 ± 0.003	2.50 ± 0.07	2.36 ± 0.06	22.78 ± 1.14	1.10 ± 0.06
10	*Lemna minor*	10C	3.40 ± 0.04	0.260 ± 0.002	0.273 ± 0.01	2.67 ± 0.01	2.68 ± 0.01	15.86 ± 0.01	1.40 ± 0.01
11	*Lemna minor*	11C ^*^	2.96 ± 0.02 (+)	0.373 ± 0.017 (+)	0.563 ± 0.01	1.87 ± 0.08 (+)	1.23 ± 0.03	37.09 ± 4.27 (+)	2.59 ± 0.23
12	*Lemna minor*	12W	4.97 ± 0.01	0.293 ± 0.014	0.302 ± 0.02	2.38 ± 0.12	2.31 ± 0.12	20.31± 2	1.35 ± 0.14
13	*Lemna minor*	13C	4.43 ± 0.01	0.306 ± 0.007	0.191 ± 0.01	2.27 ± 0.05	3.63 ± 0.14	23.77 ± 1.14	2.25 ± 0.11
14	*Lemna minor*	14BM	4.04 ± 0.01	0.301 ± 0.003	0.258 ± 0.01	2.23 ± 0.03	2.71 ± 0.12	29.44 ± 0.71	3.24 ± 0.26
15	*Lemna minor*	15C	4.97 ± 0.02	0.350 ± 0.005	0.369 ± 0.02	1.98 ± 0.03	1.90 ± 0.12	25.25 ± 0.85	2.74 ± 0.43
16	*Lemna minor*	16C	4.43 ± 0.01	0.326 ± 0.002	0.489 ± 0.01	2.12 ± 0.01	1.42 ± 0.04	26.24 ± 0.28	1.55 ± 0.14
18	*Lemna minor*	18C	4.68 ± 0.11	0.287 ± 0.029	0.326 ± 0.03	2.40 ± 0.30	2.18 ± 0.19	19.40 ± 0.24	1.35 ± 0.26
19	*Lemna minor*	19C	4.61 ± 0.45	0.234 ± 0.015	0.216 ± 0.02	3 ± 0.19	3.26 ± 0.24	21.79 ± 2.28	1.85 ± 0.20
21	*Lemna minor*	21BM	5.07 ± 0.27	0.256 ± 0.006	0.327 ± 0.02	2.72 ± 0.07	2.14 ± 0.10	26.73 ± 1.14	1.10 ± 0.12 (-)
22	*Lemna minor*	22a	3.78 ± 0.26	0.317 ± 0.019	0.512 ± 0.01	2.21 ± 0.13	1.36 ± 0.04	35.60 ± 4.55	3.64 ± 0.37
23	*Lemna minor*	23C	3.96 ± 0.19	0.260 ± 0.009	0.612 ± 0.01	2.68 ± 0.09	1.15 ± 0.01	33.14 ± 1.99	8.21 ± 0.85 (+)
24	*Lemna minor*	24W	5.07 ± 0.27	0.229 ± 0.007	0.654 ± 0.03 (+)	3.03 ± 0.09	1.06 ± 0.01 (+)	17.35 ± 0.86	1.95 ± 0.03
25	*Lemna minor*	25C	2.97 ± 0.02	0.175 ± 0.002	0.406 ± 0.07	3.95 ± 0.05	1.90 ± 0.35	20.31 ± 0.29	4.59 ± 0.34
26	*Lemna minor*	26W	5.07 ± 0.27	0.094 ± 0.010 (-)	0.229 ± 0.01	7.62 ± 0.84 (-)	3.04 ± 0.13	7.95 ± 0.57 (-)	2.14 ± 0.14
	*Lemna minor*	mean	4.38 ± 0.05	0.274 ± 0.002	0.349 ± 0.02	2.77 ± 0.04	2.31 ± 0.03	24.61 ± 0.42	3.70 ± 0.05
17	*Lemna turionifera*	17AM	9.57 ± 0.03	0.196 ± 0.033	0.199 ± 0.03	3.36 ± 0.36	3.78 ± 0.61	7.20 ± 1.57	0.95 ± 0.20
20	*Lemna toriunifera*	20AM	4.22 ± 0.30	0.433 ± 0.003	0.628 ± 0.02	1.60 ± 0.01	1.11 ± 0.04	14.38 ± 0.29	0.85 ± 0.14
	*Lemna turionifera*	mean	6.90 ± 0.13	0.314 ± 0.018	0.413 ± 0.02	2.48 ± 0.19	2.45 ± 0.28	10.79 ± 0.93	0.90 ± 0.03
27	*Lemna gibba*	1LA	2.50 ± 0.01 (-)	0.274 ± 0.002 (-)	0.329 ± 0.01 (-)	2.53 ± 0.02 (-)	2.11 ± 0.02	60.49 ± 0.99 (-)	3.98 ± 0.01 (-)
28	*Lemna gibba*	2S *	2.16 ± 0.04	0.393 ± 0.011 (+)	0.376 ± 0.01	1.77 ± 0.05 (+)	1.85 ± 0.06	108.9 ± 7.94 (+)	6.96 ± 0.57 (+)
29	*Lemna gibba*	3g	2.16 ± 0.07	0.379 ± 0.011	0.391 ± 0.03	1.83 ± 0.05	1.76 ± 0.06	69.10 ± 5.11	4.68 ± 0.40
30	*Lemna gibba*	4LA	2.17 ± 0.03	0.355 ± 0.002	0.338 ± 0.01	1.95 ± 0.01	2.05 ± 0.02	97.13 ± 1.42	6.36 ± 0.11
31	*Lemna gibba*	5S	2.35 ± 0.02	0.306 ± 0.004	0.403 ± 0.01	2.26 ± 0.03	1.72 ± 0.02 (+)	75.98 ± 2.27	5.02 ± 0.20
32	*Lemna gibba*	6LA	2.14 ± 0.01 (+)	0.321 ± 0.002	0.333 ± 0.03	2.16 ± 0.01	2.15 ± 0.01 (-)	93.20 ± 1.42	6.66 ± 0.06
33	*Lemna gibba*	7S	2.48 ± 0.05	0.298 ± 0.007	0.420 ± 0.02 (+)	2.33 ± 0.06	1.80 ± 0.01	60.73 ± 3.13	5.92 ± 0.03
34	*Lemna gibba*	8S	2.16 ± 0.01	0.333 ± 0.003	0.412 ± 0.01	2.08 ± 0.01	1.77 ± 0.02	93.10 ± 0.01	6.17 ± 0.06
	*Lemna gibba*	mean	2.26 ± 0.01	0.332 ± 0.003	0.375 ± 0.01	2.12 ± 0.01	1.90 ± 0.02	82.34 ± 1.65	5.72 ± 0.13
35	*Spirodela polyrhiza*	1AM	7.22 ± 0.93 (-)	0.323 ± 0.011	0.425 ± 0.04	2.16 ± 0.08	1.63 ± 0.02	28.70 ± 2.28	2.74 ± 0.09 (-)
36	*Spirodela polyrhiza*	2AM *	2.84 ± 0.02	0.472 ± 0.003 (+)	0.498 ± 0.05	1.47 ± 0.01 (+)	1.43 ± 0.14	59.25 ± 1.14 (+)	10.85 ± 0.03
37	*Spirodela polyrhiza*	3LA	3.86 ± 0.23	0.379 ± 0.011	0.382 ± 0.01	1.83 ± 0.05	1.82 ± 0.06	52.36 ± 3.98	4.08 ± 0.34
38	*Spirodela polyrhiza*	4AM *	2.33 ± 0.05 (+)	0.200 ± 0.009	0.784 ± 0.02 (+)	3.48 ± 0.16	0.88 ± 0.02 (+)	40.04 ± 2.56	50.88 ± 4.83 (+)
39	*Spirodela polyrhiza*	5BM	2.93 ± 0.15	0.214 ± 0.018	0.650 ± 0.02	3.30 ± 0.27	1.07 ± 0.04	16.36 ± 2 (-)	40.07 ± 0.04
40	*Spirodela polyrhiza*	6AM	3.9 ± 0.21	0.117 ± 0.014 (-)	0.149 ± 0.07 (-)	6.16 ± 0.73 (-)	13.55 ± 6.35 (-)	17.84 ± 1.71	34.01 ± 0.01
	*Spirodela polyrhiza*	mean	3.85 ± 0.21	0.284 ± 0.002	0.481 ± 0.01	3.07 ± 0.08	3.40 ± 1.05	35.76 ± 0.47	23.78 ± 0.76

DT, doubling time (days); BA, biomass accumulation (per day); RGR, relative growth rate (per day); RY, relative yield (per week). Mean values for each species are presented in high light rows. Values are mean ± SE.

(+) indicates the highest growth rate values and (-) indicates the lowest growth rate values.

*Strains with the highest growth rate.

The mean RGR for all 40 investigated ecotypes was 0.301 per day for fresh weight, ranging from 0.094 to 0.472 per day for individual clones belonging to *L. minor* 26W and *S. polyrhiza* 2AM, respectively. Additionally, the mean RGR for dry weight was 0.435 per day with a range from 0.149 to 0.784 per day for *Spirodela* clones (**Table 4**).

**Figure 4:**
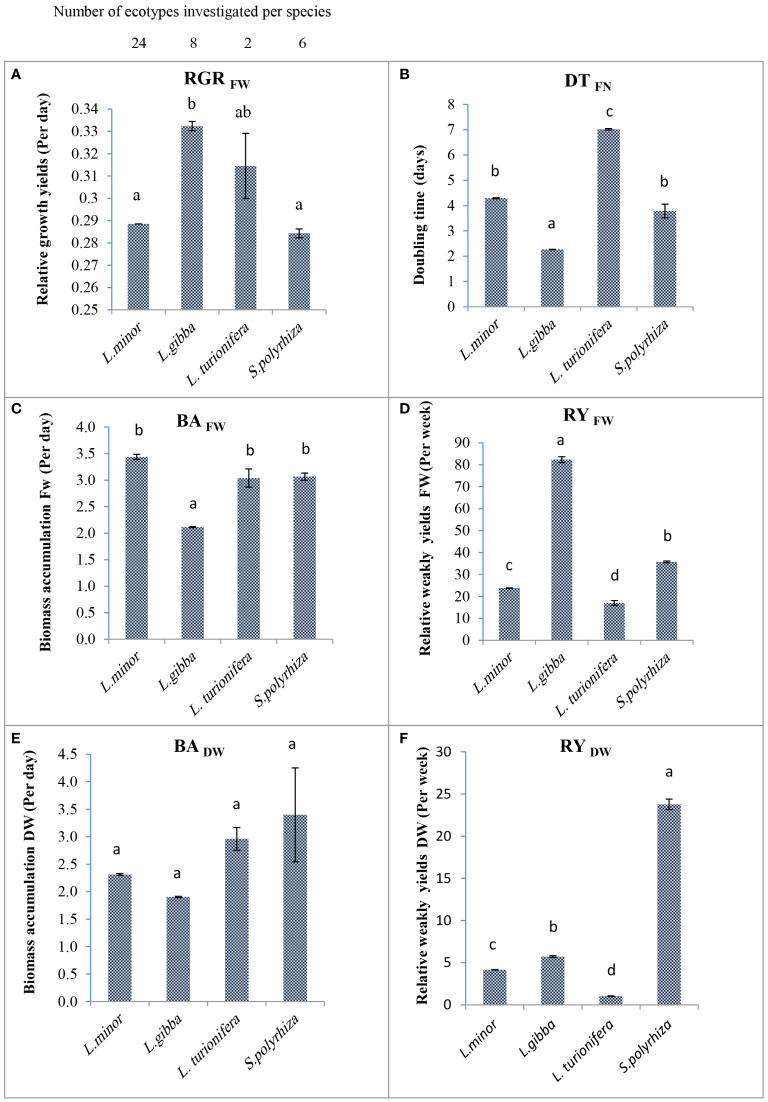
The mean fresh and dry weight–based relative growth rate (biomass accumulation, relative weekly yield, and doubling time based on frond number) of the four investigated duckweed species. **(A)** The mean RGR for the species represented by several clones. The number of clones per species is given above. **(B)** The mean value of DT_FN_ between species is significantly different. *Lemna gibba* by doubling its fronds every 2.26 days is faster than other species. **(C)** The mean BA_FW_ of four species of two genera is shown. **(D)** The maximum rate of mean RY_FW_ of four species belongs to *L. gibba* (82.3 per week). **(E)** The mean of BA_DW_ is not significantly different among species. **(F)** The mean dry weight (mg) produced after 1 week (RY) from primary inoculum among species is significantly different (*P*<.05). In all figures: The column height shows the mean growth parameters of the four investigated species. Error bar was indicated in the figures. Letters above the columns indicate significance according to ANOVA: means of columns marked with the same letter (either ‘a’ or ‘b’) do not differ to a statistically significant extent; differences are statistically significant when the columns are marked with single, different letters. The means of the columns marked with ‘ab’ do not differ significantly from means of columns marked with either ‘a’ or ‘b’.

As shown in **Figure 4A**, the mean RGR based on fresh weight for four species belonging to two duckweed genera ranged from 0.284 per day for *S. polyrhiza* to 0.332 per day for *L. gibba*. The mean RGR for *L. minor* is 0.274 per day. The RGR for *L. gibba* was significantly higher than for any other species (*P*<.05). This was due to a higher weight gain and more fronds after 7 days of cultivation. There were no significant differences between the mean RGR_FW_ of *L. minor* and *S. polyrhiza* due to a wide range of RGR values among *L. minor* clones.

The dry weight analysis gave similar results to fresh weight. Among the 24 *L. minor* clones, the highest RGR_DW_ values were obtained by clones 11C, 23C, and 24W with 0.563, 0.612, and 0.654 per day, respectively. These values were not significantly different from each other (*P*<.05). The clone with the significantly highest RGR_DW_ among the eight *L. gibba* clones was clone 7S with 0.420 per day. Among the six clones of *S. polyrhiza*, 4AM showed the highest value with RGR_DW_ = 0.784 per day. Fresh and dry weight RGR within species are significantly different (*P*<.05), especially in *L. minor* and *S. polyrhiza*. Some of the ecotypes of *L. minor* and *S. polyrhiza* studied as well as one of the clones of *L. turionifera* had an RGR higher than 0.600 per day (**Table 4**). This is a significantly higher RGR value compared with other ecotypes.

DT, which is based on the number of fronds, and BA, based on the fresh or dry weight, reflect the RGR value but numerically in the opposite way (see Equation (3) above). It indicates how much time is needed to double the number of fronds or biomass. DT based on frond number was investigated for all ecotypes. The lowest DT (rapid growth) within species was measured for *L. minor* 11C (2.96 days)*, L. gibba* 6LA (2.14 days), and *S. polyrhiza* 4AM (2.33 days), which doubled their frond number every 51 to 71 h (**Table 4**). A comparison of the mean values for DT of the investigated clones of the four species is shown in **Figure 4B**. The mean value of frond doubling time for *L. gibba* with 2.26 days is significantly lower than that of the other species (*P*<.05).

The mean biomass accumulation based on fresh weight (BA_FW_) of eight *L. gibba* clones was 2.12 days. Thus, this species had significantly higher productivity than the other species (*P*<.05; **Figure 4C**). On the other hand, the mean biomass accumulation based on dry weight (BA_DW_) of *L. gibba* is consistent with BA_FW_. However, this is not significantly different between species (**Figure 4E**). The significantly fastest BA_FW_ within *L. minor* was observed for strain 11C with 1.87 days (*P*<.05; **Table 4**). The two clones of the rare *L. turionifera* had BA rates of 1.60 and 3.78 days. To increase the accuracy of growth parameters in *L. turionifera*, it is necessary to continue the work with more ecotypes.

The RY of biomass accumulation based on fresh weight (RY_FW_) of all investigated ecotypes ranged from 108.92 (*L. gibba* 2S) to 7.2 per week (*L. turionifera* 17AM). The highest mean RY_FW_ among the species belonged to *L. gibba* (82.34 per week) and the lowest mean RY_FW_ to *Spirodela* (35.76 per week) and *L. minor* (24.61 per week). The differences among the four species were statistically significant (*P*<.05; **Figure 4D**). Analysis of the RY value showed that the results were consistent with BA and DT. In addition, intraspecies data for *L. minor* showed that the highest relative yield of 37.09 per week was obtained by *L. minor* 11C due to its better growth potential. Despite the results for mean fresh weight (**Figures 4D, F**), where *L. gibba* showed the highest RY_FW_, *S. polyrhiza* had the highest value in RY_DW_ in the species comparison with 23.78 per week (**Figure 4F**) while *L. gibba* (5.72 per week) and *L. minor* (3.70 per week) were both in the lower range.

## Discussion

In the present study, biomass production screening was performed based on growth potential analysis on native duckweed species from Iran coupled with DNA barcoding based on two standard chloroplast markers. Growth parameters were used to identify the most productive ecotypes of each of the four native duckweed species (*Spirodela polyrhiza*, *Lemna minor*, *L. gibba*, and *L. turionifera*) among the 40 clones studied. *Lemna minor* 11C, *L. gibba* 2S, and *S. polyrhiza* 4AM showed growth rates and a relative yield even higher than any terrestrial plants reported in previous studies (Ziegler et al., 2015; Koca and Erekul, 2016). These data demonstrate the importance of comprehensive studies on duckweed for biomass accumulation in biological production systems and food security. In addition, for the first time, the species *L. turionifera* was detected for Iran based on DNA barcoding analysis, and the two clones were included in the biomass screening.

Primary identification of duckweed based on morphological characters identified three species belonging to two genera: *Spirodela polyrhiza*, *Lemna minor*, and *L. gibba*. However, subsequent DNA barcoding analysis revealed a fourth species: *L. turionifera*, which is not easily distinguishable from *L. minor* due to the strong reduction in their morphology. After an appropriate literature research, *L. turionifera* was not listed for Iran in Landolt’s monograph (Landolt, 1986), nor in the online Flora of Iran (2022) (http://flora-iran.com/central-herbarium-of-tehran-university/plant-list/), nor in the two online platforms “Plants of the World Online (2022)” (https://powo.science.kew.org/) and “Global Biodiversity Information Facility – GBIF (2022)” (https://www.gbif.org/).

The variation of the studied sequences is rather low, as is known for several duckweed species (Borisjuk et al., 2015; Chen et al., 2022), but also to be expected for a DNA barcode as they should show lower genetic variation within than between species (Dasmahapatra and Mallet, 2006). Interestingly, with the exception of two accessions, all other accessions identified as *L. minor* show the same haplotype as the reference sequence of *rps16* for clone 8292 from Iran, which was already mentioned in Landolt and Urbanska-Worytkiewicz (1980) and, thus, has been kept in culture for more than 40 years, which again could indicate a relatively constant haplotype pool. However, further studies are needed to reach similar conclusions as for *S. polyrhiza*, namely, that the mutation rate in this species is very low (Ho et al., 2019; Xu et al., 2019). Because the molecular markers used do not allow us to draw conclusions about hybridization events, the possibility that the *L. minor* clones identified here may be hybrids between *L. minor* and *L. turionifera* cannot be ruled out. Both species occur in the area, and hybridization between these two species has already been demonstrated by Braglia et al. (2021).

RGR is an important factor to show physiological responses of plants to light, temperature, CO_2_, and nutrients, but its interpretation is less intuitive (Buxbaum et al., 2022). Therefore, it is often mathematically transformed and reported as BA or RY, which are common parameters for large-scale screening of plant growth potential (Ziegler et al., 2015). Fast-growing species show higher RGR under standard cultivation conditions. Higher RGR is due to efficient nutrient and CO_2_ uptake in fronds (Naumann et al., 2007; Ziegler et al., 2015; Ghanem et al., 2019). High RGR causes duckweed to rapidly double its frond number and biomass, which increases its photosynthetically active surface area per unit area. One of the most important variations is the nutrient medium. Many studies demonstrate that duckweed exhibits optimal growth potential and accumulates more biomass in species-specific nutrient media (Kittiwongwattana and Vuttipongchaikij, 2013; Muranaka et al., 2015; Ghanem et al., 2019). This study was the first to use a nutrient medium optimized for growth for each species (data in preparation).

In this study, a relative linear relationship between RGR, DT or BA, and RY was investigated (**Figure 4**). In addition, growth factors based on dry weight, especially RY_DW_, are more reliable parameters to study the stability of biomass gain in the duckweed family. Approximately 92%–94% of the fresh weight of duckweed consist of water, which is lost after drying (Pagliuso et al., 2022). This is confirmed by the relatively low yield (RY_DW_) results for *L. gibba* shown in **Figure 4F**. Despite the highest mean RY_FW_ value (**Figure 4D, F**) for *L. gibba*, almost 90% of the fresh weight was lost to drying. In contrast, for *Spirodela*, only 35% of the fresh weight was lost to drying, and on average, 65% of the weight was retained as dried biomass (**Table 4** and **Figure 4F**).

Based on the analysis of growth potential of 40 ecotypes, ecotypes with better growth potential under standard growing conditions were identified. *Spirodela polyrhiza* and *L. gibba* showed higher average growth factors among the studied species in this experiment. On the other hand, *L. minor*, the most widespread and easily manipulated species, with ecotype 11C provided one of the most productive duckweed clones in this experiment for future research. Considering that growth potential is shown to have a wide range of values within genera and species and, thus, significant differences among clones and ecotypes, it is concluded that species or genus specificity is not a reliable method for screening growth potential. In other words, growth parameters determined for one species or genus cannot be generalized to all clones of a species. This is consistent with Bergmann et al. (2000), who suggest focusing on the geographic isolate (ecotypes) rather than the species level. Ziegler et al. (2015) also confirm that screening on the ecotype level is the most reliable method for screening growth factors in duckweed. We found three ecotypes with high growth potential such as *S. polyrhiza* 2AM with an RGR_FW_ of 0.472 per day, which is higher than the value reported by Ziegler et al. (2015), ranging from 0.168 to 0.386 per day for seven *S. polyrhiza* clones. Other dependent parameters, such as BA_FW_ (1.47 per day) and RY_FW_ (59.25 per week), are consistent with those reported in the literature (Ziegler et al., 2015). In addition*, L. gibba* 2S was found to be the fastest ecotype in biomass accumulation with 1.77 per day. Similarly, the relative yield after 7 days of inoculation is the highest value (108.92 per week) for *L. gibba* 2S, which is consistent with those from previous reports (Ziegler et al., 2015). It is noteworthy that *L. gibba* already has a good potential to gain biomass in a short time. This is due to the rapid proliferation rate and gibbous fronds in this species. As mentioned earlier, under unsuitable culture conditions, *L. gibba* loses its gibbosity and looks like *L. minor*. NF is the best culture medium for *L. gibba* to form gibbous fronds and increase the rate of biomass accumulation (Muranaka et al., 2015). In addition, the widely distributed duckweed species *L. minor* with ecotype 11C has an RGR_FW_ value of 0.373 per day. This is comparable to the value of RGR_FW_ = 0.422 per day reported by Chakrabarti et al. (2018), where *L. minor* was cultivated with high-efficiency organic manure. The RGR_DW_ of *L. minor* 11C was 0.563 per day, which was among the highest values in the study, while Petersen et al. (2021) obtained a maximum RGR_DW_ of 0.23 per day for *L. minor* under nonsterile conditions and with high-yielding agricultural fertilizer treatment. Despite the different cultivation conditions, the highly concentrated nitrate-N medium and light intensity (270 μmol/m^2^/s higher than in the present study) were effective factors for optimal growth.

Duckweed is described as one of the fastest angiosperms due to its ability to double its biomass in a short time period. Demmig-Adams et al. (2022) suggest that the rapid growth of duckweed, even under limited light conditions, may be related to relatively thin photosynthetic organs (fronds) without complex structures on the water surface, allowing all chloroplasts to be involved in sugar production. The free-floating fronds with high availability of nutrient resources have higher photosynthetic yields. Terrestrial plants, on the other hand, use a significant amount of sugar to build the complex structures of their stems, leaves, and roots, which is a time-consuming process that involves the production of some organs that are unusable for human nutrition. The presented results shown in **Table 4** confirm the superiority of growth rate of duckweeds by comparing their RGR with the RGR of crops. Of course, plant species differ greatly in their relative growth rate even when compared under similar environmental conditions (Tomlinson et al., 2014). However, duckweed is shown to have a higher RGR_DW_ compared with many crops despite its reduced and leaf-like structure that makes it one of the lightweights among crops. Poorter (1989) reports RGR_DW_ of eight herbaceous wild species under optimized growth conditions with the highest RGR value of 0.268 per day for *Urtica dioica*. Potter and Jones (1977) report RGR_DW_ after 28 days for nine species of important crops with a value ranging from 0.202 per day for soybean to 0.391 per day for sorghum. In contrast, an RGR value of 0.255 per day was determined for maize under the best experimental conditions. The data presented in **Table 4** and **Figure 4** show that most of the duckweed clones that were studied had an RGR based on fresh and dry weight (and corresponding RY) that was higher than the 0.255 per day described for maize. Overall, the superior growth characteristics and short harvest time of duckweed compared with crops may lead to higher biomass and economic production.

As reported by the Food and Agriculture Organization of the United Nations (FAO), food security is one of the most important challenges in the world (https://www.fao.org/state-of-food-security-nutrition/2021). Due to the increase in world population, reduction in food resources, depletion of nutrients in soils, and global climate change leading to a reduction in crop production, it is imperative to pay more attention to alternatives with low water and soil requirements that are cost-efficient, have short harvesting times, and produce more biomass. Duckweed as an aquatic crop has several advantages over terrestrial crops: it absorbs nutrients directly from water, is easy to grow and harvest, has low water requirements (due to its short growing season), and does not compete with crops in agricultural land use, allowing for higher biomass production per hectare even in dry areas (Toyama et al., 2017; Tursi, 2019). To date, interest in large-scale cultivation of duckweed in greenhouses and under protective structures has grown through commercial companies, such as LENTEIN™ (https://www.parabel.com/) and Rubisco Foods (2022) (https://rubiscofoods.com/).

## Conclusion

The results of this study show that three selected ecotypes of *Lemna* and *Spirodela* species can provide high yields of fresh and dry biomass under optimal growth conditions. The data confirm that most ecotypes of duckweed can grow faster than traditional crop plants. However, this high RGR obtained under the optimized culture conditions for the selected ecotypes may be different under real environmental conditions. However, the fact that duckweed as an emergent crop has a high relative yield and accumulates more biomass in a short time was also confirmed under agricultural cultivation conditions. The data in this study illustrate the numerous potentials of the selected duckweed ecotypes for commercial biomass production and biotechnological application. However, several strategies are needed to optimize duckweed growth with low cost, simplicity, and scalability.

## Data availability statement

The datasets presented in this study can be found in online repositories. The names of the repository/repositories and accession number(s) can be found in the article/**Supplementary Material**.

## Author contributions

AS is the executor of plan, ET is PhD student and project manager, MB contributed to DNA barcoding analysis, FF, SS, NR and MA cooperated in biomass measurements, MJ organized the database. All authors contributed to manuscript revision, read, and approved the submitted version.

## Funding

This research was supported partially by grants from Iran National Science Foundation (INSF) (97024978), Center for International Scientific Studies and Collaboration (CISSC) (990160) and National Institute of Genetic Engineering and Biotechnology (NIGEB) (980301-II-714).

## Conflict of interest

The authors declare that the research was conducted in the absence of any commercial or financial relationships that could be construed as a potential conflict of interest.

## Publisher’s note

All claims expressed in this article are solely those of the authors and do not necessarily represent those of their affiliated organizations, or those of the publisher, the editors and the reviewers. Any product that may be evaluated in this article, or claim that may be made by its manufacturer, is not guaranteed or endorsed by the publisher.
