# Supplementary figures and images for: DNA barcoding and biomass accumulation rates of native Iranian duckweed species for biotechnological applications
Source: Front Plant Sci. 2022 Nov 29;13:1034238. doi: 10.3389/fpls.2022.1034238 (PMC9744944; doi:10.3389/fpls.2022.1034238)

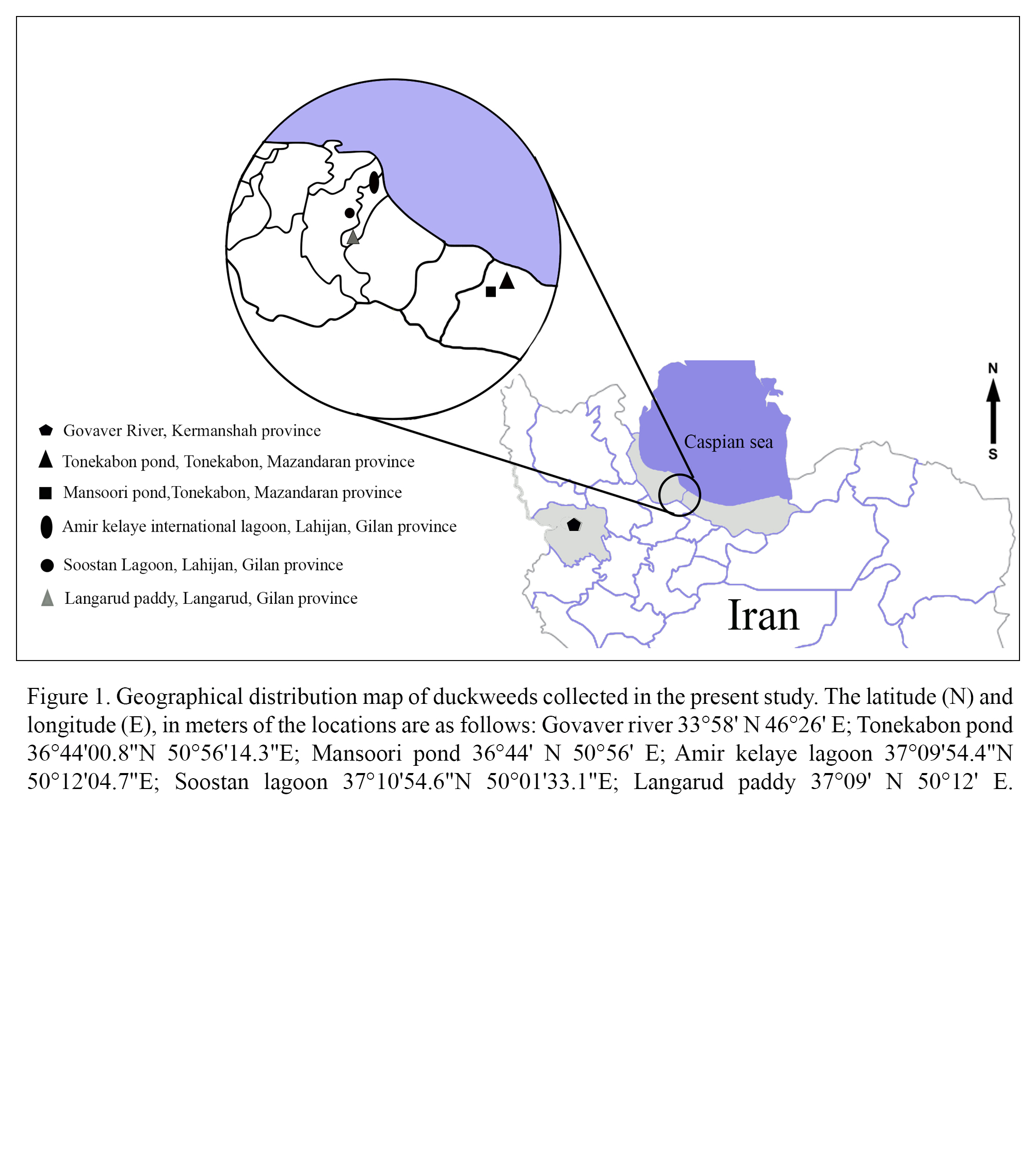

Supplement: Supplementary file 1 [file Image_1.jpeg]

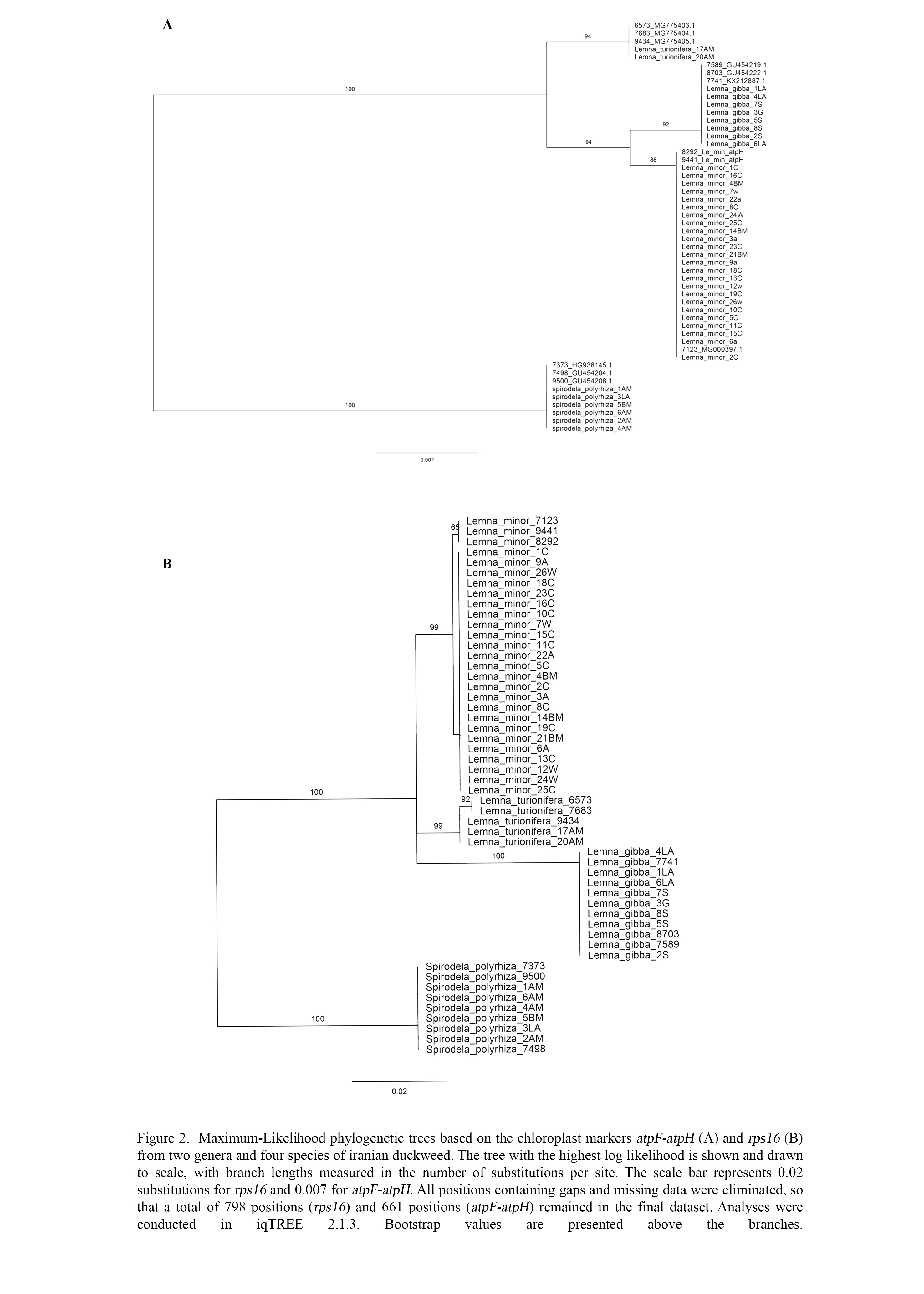

Supplement: Supplementary file 2 [file Image_2.jpg]
